# Unraveling Natural Killer T-Cells Development

**DOI:** 10.3389/fimmu.2017.01950

**Published:** 2018-01-09

**Authors:** Sabrina Bianca Bennstein

**Affiliations:** ^1^Nuffield Department of Surgical Sciences, John Radcliffe Hospital, University of Oxford, Oxford, United Kingdom

**Keywords:** invariant NKT cells, natural killer T cells, natural killer T type II cells, natural killer T development, natural killer T lineage, natural killer T subsets

## Abstract

Natural killer T-cells are a subset of innate-like T-cells with the ability to bridge innate and adaptive immunity. There is great interest in harnessing these cells to improve tumor therapy; however, greater understanding of invariant NKT (iNKT) cell biology is needed. The first step is to learn more about NKT development within the thymus. Recent studies suggest lineage separation of murine iNKT cells into iNKT1, iNKT2, and iNKT17 cells instead of shared developmental stages. This review will focus on these new studies and will discuss the evidence for lineage separation in contrast to shared developmental stages. The author will also highlight the classifications of murine iNKT cells according to identified transcription factors and cytokine production, and will discuss transcriptional and posttranscriptional regulations, and the role of mammalian target of rapamycin. Finally, the importance of these findings for human cancer therapy will be briefly discussed.

## Introduction

Natural killer T-cells belong to the T lymphocyte family and are found in many different tissues within the body (1). Unlike conventional T lymphocytes (convT cells), the rearranged T-cell receptor (TCR) of NKT cells recognizes lipid antigens presented on CD1d, a “major histocompatibility complex (MHC)-like molecule,” instead of MHC itself (1), giving them an adaptive characteristic (2). Their tissue localization is driven by chemokine receptors expression, such as CXCR3 (driving accumulation in inflamed tissues) and CXCR6 (important for liver homing) (3). NKT cells were shown to rapidly produce cytokines after stimulation, which is an innate-like feature. Thus, they are considered to “bridge innate and adaptive immunity” (2). This enhances the recruitment of innate-like cells (4), DC, and B-cell maturation (5).

Natural killer T-cells are divided into two groups according to their TCR chains. Type I NKT cells, also called invariant NKT (iNKT) cells, use a distinct invariant TCR α-chain with limited TCR β-chain repertoires, while Type II NKT (NKT_II) cells express broad ranges of different TCR chain combinations (6). Evidence suggests the existence of NKT-like cells, such as other CD1-restricted T-cells and MR1-restricted mucosal-associated invariant T-cells cells (7), but these populations will not be discussed in this review.

Antigen recognition by NKT cells and their development within the murine thymus will be discussed. Recent publications suggest a classification of murine iNKT lineages according to their transcription factor (TF) expression and cytokine secretion. Therefore, the author will discuss transcriptional and posttranscriptional regulation of iNKT cell development and function, and the role of Mammalian Target of Rapamycin (mTOR) within iNKT cell subsets. This new lineage concept will be compared to the previous categorization into three developmental stages.

## iNKT and NKT_II Cell Antigen Recognition

Unlike convT cells, iNKT cells bear a semi-invariant TCR, upon rearrangement of a single TCR α chain with a unique Jα segment, in combination with limited TCR β-chains usage. This results in a rearranged Vα14-Jα18/Vβ8, Vβ7, or Vβ2 TCR in mice and Vα24-Jα18/Vβ11 in humans (1). Human iNKT cells can be divided into CD4^+^, CD8^+^, and CD4^−^CD8^−^ subsets; and murine iNKT cells into CD4^+^ and CD4^−^CD8^−^ (7). This TCR shows unique reactivity to the glycolipid αGalCer bound to CD1d (8), and CD1d-αGC tetramers have proven an invaluable tool to study iNKT cell biology (9). Conversely, NKT_II cells use different combinations of TCR chains, both in mice and humans. Due to their diverse TCR rearrangement, one possibility to study murine NKT_II cells is by comparing mice lacking only iNKT cells [*J*α*18*-deficiency (10, 11)] with mice lacking all NKT cells [*Cd1d-*deficiency (12)]. Using a Jα18-deficient interleukin (IL)-4 reporter model, type II NKT cells can be tracked by their expression of GFP and TCRβ (13). This model has allowed to demonstrate that murine NKT_II cells display diverse α- and β-chains with dominant Vα8 and Vβ8.1/8.2 chains (13). Even though NKT_II are dominant in humans (6), due to their TCR chain diversity and the lack of specific reagents to identify them, they have not been studied as intensively as iNKT cells. Thus, many details of NKT_II subsets are ill defined. What is currently known about NKT_II cells has been recently reviewed (14) and will not be further discussed within this review.

Both NKT cell types share the recognition of various lipid antigens presented on CD1d molecules (1), but use different complementarity-determining regions loops for antigen binding (6). Like convT-cells, NKT cell types are selected within the thymus (1).

## Overview Over the Lineage Fate within the Murine Thymus

CD4^−^CD8^−^ lymphoid precursors travel from bone marrow *via* blood to the thymic corticomedullary junction (15). Due to the close contact with thymic epithelial cells and mechanisms, which will not be discussed in this review, the “thymocytes commit to a T-cell fate” with TCR rearrangement and upregulation of CD4 and CD8 (15). At this stage, the NKT cell population seems to split from convT-cells (7). iNKT cells are selected if their TCR recognizes self- or foreign lipid antigens on CD1d molecules expressed by CD8^+^CD4^+^ thymocytes [double positive (DP)] (16). Furthermore, iNKT cell development needs the expression of NFKB-activating protein and histone deacetylase 3 (17) and depends on microRNAs (18, 19). As the Jα18 rearrangement is a late event, DP cells need to survive a distinct period of time. Thus, all mutations limiting the lifespan of DP cells affect iNKT development (20).

Further differentiation and maturation of CD69^+^CD24^+^ iNKT precursor cells is initiated by parallel binding to the co-stimulatory signaling lymphocytic activation molecules (SLAMs), SLAMF1, and SLAMF6, which signal downstream *via* the SLAM-associated protein (SAP) (21). SLAMF6 augments downstream phosphorylation due to enhanced TCR signaling, increasing the expression of the TF *Erg2* (22). iNKT cells were also shown to receive stronger TCR signaling compared to convT-cells (23). Interestingly, stimulation by the convT-cell co-stimulatory molecule CD28 induced only a minor increase in *Erg2* expression (22). ERG2 binds to the *Zbtb16* promoter region, which induces the expression of the TF promyelocytic leukemia zinc finger (PLZF) (22), a master regulator of iNKT cell development and function (24).

*Zbtb16*-deficient mice are unable to develop iNKT and NKT_II cells further than the naïve state (13, 24), showing the importance of PLFZ in early NKT development. In line with these findings, SAP-deficient mice show a decrease in PLZF expression in early developmental stages in iNKT cells (25) and decreasing NKT_II numbers by 10-fold (13). In this early developmental state [which was originally defined as stage 0 (1)], NKT cells express the surface molecules CD69^+^CD24^+^CD4^+^CD8^+/−^ (1, 13) and express the TFs ERG2, and PLFZ.

## The Developmental Stages of Murine iNKT Cells

**Figure 1:**
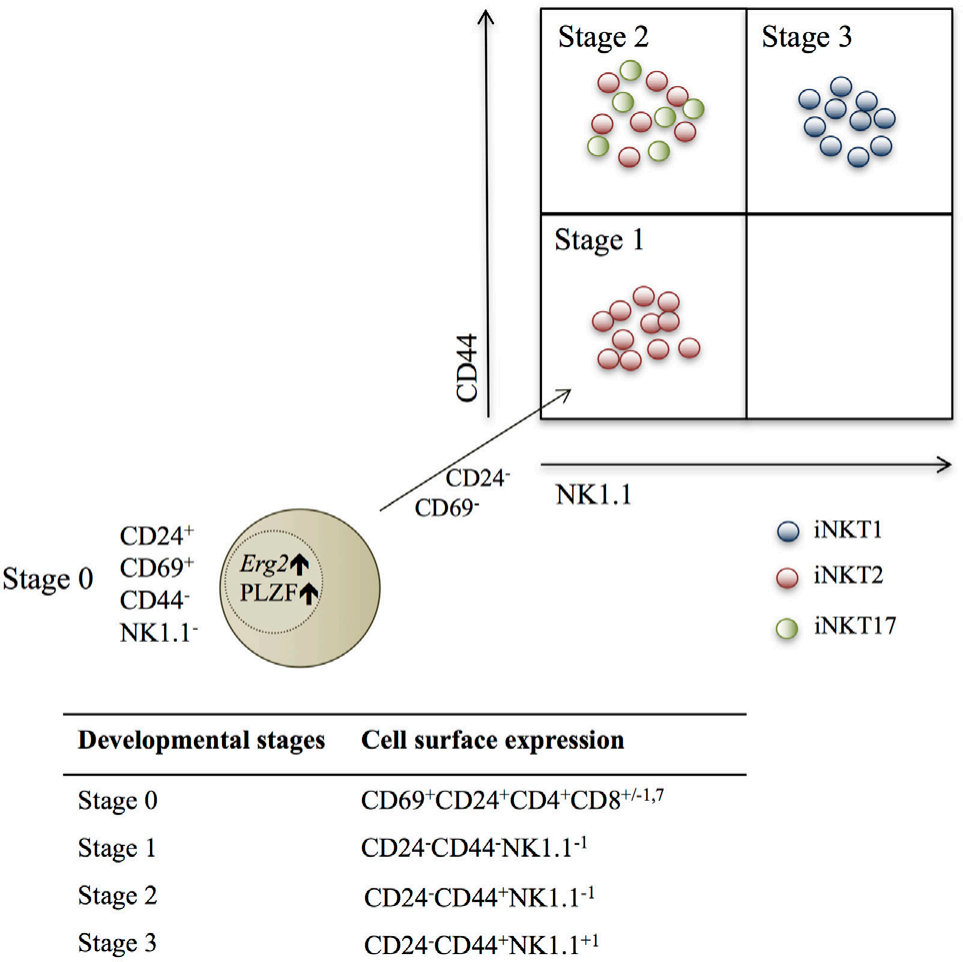
Schematic fluorescence-activated cell scanning plot depicting the three identified invariant NKT (iNKT) subsets within the described developmental stages, according to NK1.1 and CD44 expression. Red dots are iNKT2 cells, green dots are iNKT17 cells, and blue dots are iNKT1 cells. The beige dot represents a stage 0 iNKT cell, which expresses the transcription factors Erg2 and PLZF, and decreases CD24 and CD69 expression during the development into stage 1 NKT cells (26, 28, 29).

Three developmental iNKT stages based on cell surface molecule expression of CD44 and NK1.1 have been described (Figure 1). However, this categorization is not ideal, as NK1.1 is not universally expressed in all mouse strains (26, 27). Recently, iNKT cells were categorized according to TF and cytokine expression profiles into iNKT1, iNKT2, and iNKT17 lineages (26–28), and these were mapped into the developmental stages (26, 28, 29) (Figure 1).

The new classification of iNKT cells alternative to the shared developmental stages favors clear lineage separation (27, 28, 30). This review will give more insight into the newly defined iNKT lineages and will discuss the relationship between the three groups in relation to the developmental stages. Of note, evidence of more iNKT subsets exists (2).

## Transcriptome Analyses of iNKT1, iNKT2, and iNKT17 Cells

The categorization of iNKT subsets was done *via* intracellular staining and subsequent sorting according to the TFs: *T-bet* for iNKT1 (31), GATA binding protein 3 (*Gata-3*) for iNKT2, and *Ror*γ*t* for iNKT17 (26–28, 31). Parallel experiments were based on *Zbtb16* as *Gata-3* equivalent (27, 31).

Using this method, transcriptome analyses showed three distinct populations in principle component analyses (PCA) (28, 31). Using several RNA sequencing methods, one study identified unique homing molecules within individual iNKT subsets in C57Bl/6 mice: CXCR3, CCR5, and VLA-1 for iNKT1, CCR4, and CCR9 for iNKT2, and CCR6, *Itgb4, Itgb5*, and *Itgb7* (encoding for integrin subunits) for iNKT17 (31), which may explain their difference in tissue distribution and corresponding altered cytokine profile of the three subsets (32). In a different paper, the Hogquist group used RNA sequencing and microarray data from Balb/c and C57Bl/6 mice to investigate the relationship between the above described iNKT cells with other cell subsets including innate lymphoid cells (ILCs), T-cells, and natural killer (NK) cells (28). The iNKT1 transcriptome was similar to T_H_1, ILC1, γδ T-cells, and NK cells (28), which also express IFNγ. iNKT2, and iNKT17 showed more transcriptome similarity to their “respective ILC and γδ T-cell counterpart,” but not to T_H_2 and T_H_17 (28). As ILC precursors express PLZF (33), the authors suggested PLZF as master TF for innate like T-cells and ILCs (28), indicating a more “unidirectional gene programming in IFNγ expressing cells” (28). It would have been interesting to know if the authors found other possible interesting regulatory genes, as they only acknowledged already described genes for the three different iNKT populations, yet, these genes did not show the highest fold change within the volcano plots.

### Transcriptional Regulation of iNKT1 Cells

**Figure 2:**
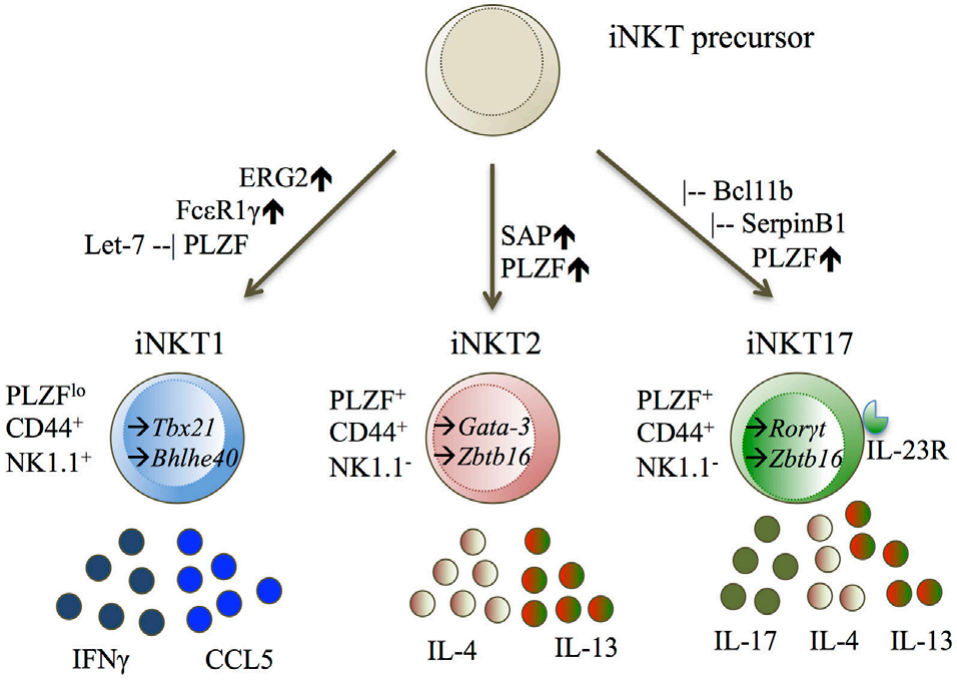
iNKT1, iNKT2, and iNKT17 displayed with their transcription factors (TF), cell surface molecules, and cytokine secretion. Diagram legends: – inhibiting, ↑ upregulated, → expressed TF (25–29, 34, 35, 41).

So far, the iNKT1 subset has been defined by the upregulation of *T-bet* (*Tbx21*) (26–28, 31), *Erg2* (34), FcεR1γ (27), and the microRNA *let-7* (29). iNKT1 cells express the cytokines IFNγ (26, 27, 31) and CCL5 (27, 31) (Figure 2).

In order to produce IFNγ, *T-bet* and its co-factor Bhlhe40, which opens the *Ifg*γ locus, are needed (35). Besides a crucial role in early iNKT development, *Erg2* expression also seems to be essential for further iNKT1 development. *Erg2*-deficient thymocytes do not develop past developmental stage 2 (34). Besides binding to the *Zbtb16* promoter (22), *Erg2* can bind to the *Il2rb* promoter (34), inducing the expression of CD122, a shared component of the IL2R (36) and IL-15R (29, 36). The responsiveness to IL-15 is needed for final development into stage 3 NKT cells (34). As only iNKT1 cells were described to belong into this stage, the signaling *via* IL-15 could lead to downstream cell intrinsic restructuring programs favoring an iNKT1 fate. In favor of this hypothesis is the demonstration that IL-15 signaling regulates *T-bet* in murine CD8αα^+^ intraepithelial lymphocytes (37). Whether this also applies to iNKT cells remains to be elucidated. CD14^+^ monocytes/macrophages, and to some extent B cells, were shown to produce IL-15 within the medulla and in cortical clusters within human thymi (38). This might be the source of IL-15 for iNKT1 cells. Another control mechanism is the upregulation of the microRNA *let-7*, which leads to a downregulation of PLFZ as “two conserved binding sides were found in the 3′UTR” of *Zbtb16* (29). Further, the mRNA expression profiles of *Zbtb16* and *let-7* showed inverse correlation (29). Interestingly, this paper showed conserved let-7 binding sides in mice and human, leading to the question if *let-7* is also regulating expression profiles in human iNKT cells. However, a downregulation of PLZF in iNKT1 cells was only shown within the thymus (29), proposing the role of other mechanisms in peripheral tissues (29). Additionally, NKT subtypes might also be selected *via* their TCR signaling capacity, as *Fc*ε*R1*γ-deficient mice showed a decreased iNKT1 cell count, but an increase in iNKT2 cells (27). An upregulation of the *Fc*ε*R1*γ chain, generally known as part of the high-affinity IgE receptor, was detectable in iNKT1 cells (27). Together with CD3ζ, the *Fc*ε*R1*γ chain can form the natural cytotoxicity receptor NKp46 (39), first described in NK cells, while in T-cells, it could lead to an altered TCR signaling (27).

### Transcriptional Regulation of iNKT2 and iNKT17 Cells

The iNKT2 and iNKT17 cell subsets cannot be easily separated from one another. iNKT2 cells were defined by literature to upregulate either *Gata-3* (26), *Irf-4* (26, 31), or *Zbtb16* (27, 31), and expressing the cytokines IL-4 (26, 27, 31) and IL-13 (27, 31). iNKT17 cells are defined by upregulation of *Ror*γ*t* (25–27, 31), IL-23R (27, 31), SerpinB1 (27, 31), Bcl11b (40), and expression of the cytokine IL-17 (27, 31) (Figure 2).

By cell surface molecule classification, iNKT2 cells are thought to belong to developmental stage 1 and 2, sharing stage 2 with iNKT17 cells (26) (Figure 2). iNKT2 and iNKT17 cells were also shown to share gene expression patterns (27), including *Gata-3* (26), *Irf-4* (26), and *Zbtb16* (27). It is difficult to judge if these findings are universal, as one paper has a limiting statistical power of two, but uses Balb/c and C57Bl/6, while the other paper shows exclusively C57Bl/6 data.

A recent publication highlights the possible importance of SAP for driving an iNKT2 fate. SAP-deficient mice showed decreased expression of *Gata-3* and *Zbtb16*, but an increase of *Ror*γ*t* leading to 10-fold more iNKT17 cells in these mice (25). Hardly any difference in iNKT1 cell count or percentage was detectable (25).

The serine protease SerpinB1 is associated with regulation of T_H_17 and IL-17-producing γδ T-cells (41). Interestingly, *SerpinB1*-deficient mice showed a percentile increase of iNKT17 cells, even though total iNKT cells numbers remained unchanged (27), leading to the authors’ conclusion that SerpinB1 is a negative regulator for IL-17 producing cells (27, 31). Another regulatory TF for iNKT17 cells could be Bcl11b, as PLFZ^cre^Bcl11b^fl/fl^ mice showed an overall reduction in iNKT cells. This was due to reduced survival, with a higher percentage of cells within stage 0–2 and a reduced stage 3 subset (40). By analyzing the specific TFs and cytokine secretion, these mice showed reduced *T-bet* and IFNγ expression and reduced IL-4 expression, but similar *Gata-3* expression compared to WT (40). Simultaneously, Rorγt and other iNKT17-associated genes, which were found exclusively expressed on iNKT17 cells (31), were upregulated not only in iNKT17 cells but also in iNKT2 and iNKT1 (40).

### Cross Antagonism in iNKT Cells

Initially, it seems contradicting that only iNKT2 cells are affected by decreased PLZF expression, as iNKT17 and iNKT2 cells are thought to express the same developmental stage surface molecules and were both shown to express PLZF. High expression of PLZF might not be mandatory for iNKT17 differentiation, but may be needed for iNKT2 and iNKT17 to separate from an iNKT1 fate, as mature iNKT1 cells show low PLZF expression. In favor of this is the cross antagonism of T_H_1 and T_H_2 (42), where *Gata-3* and *T-bet* can inhibit one another and decide the cell fate (42). However, evidence is growing against this assumption, as lineages have been shown to not necessarily arise from precursors, but can arise from “direct conversion” from one type to another through genetic reprogramming (43) and due to “poised” epigenetic stages (44). This might explain why, in developmental stages 2 and 3, a co-expression of *Ifn*γ and *Il-4* mRNA was detected (29) and *Tbx21—*the gene for T-bet and CXCR3 can be found in iNKT2 cells (31).

As an antagonism of *Gata-3* and *Ror*γ*t* has not been reported yet, there is the possibility that iNKT2 and iNKT17 cells cannot be seen as two separate populations. It could be possible that iNKT17 cells can convert into iNKT2 cells depending on the microenvironment as suggested by Waddington’s epigenetic landscape in 1957. This would explain their shared genetic program and developmental stage surface molecules. Transcriptome analyses support this as *Gata-3* expression was not unique to iNKT2 cells and could also be found in iNKT1 and iNKT17 cells (27, 28). However, only iNKT2 cells were shown to secrete IL-4 (26, 27). As *Gata-3* is seen as the regulatory TF for IL-4 expression and T_H_-like lineage fate (26), posttranscriptional regulation must be present to inhibit *Gata-3* from binding to the IL-4 promoter region. Two recent papers suggest a role in microRNAs to control *Gata-3* expression. The “genetic variant rs1058240” and “microRNA-720 are proposed to bind to human *Gata-3* 3′UTR” (45, 46). The overexpression of microRNA-720 leads to a reduced expression of *Gata-3* mRNA and protein levels as well as to a decrease of surface molecules associated with human alternative macrophage activation (46). However, the authors did not study the effect of the reduced *Gata-3* expression in respect to IL-4 expression. Further evidence might emerge from analyses of the epigenetic status of the lineage regulatory genes within convT-cells (44). T_H_17 cells express majorly permissive H3K4me3 at the *Gata-3* locus, thus T_H_17 cells might be able to convert into T_H_2 cells (44), while T_H_2 cells show repressive H3K27me3 marks at the *Ror*γ*t* locus (44). Even though this needs to be validated within iNKT cells, it might explain why only negative regulators have been found to give rise to iNKT17 cells.

Interestingly, the deficiency of *Runx1* (47) and *c-Maf* (48), which have been identified to be expressed in all three iNKT subsets, can lead to selective impairment of iNKT17 differentiation. *Runx1* is essential for overall iNKT development, proliferation, and survival (47), while *c-Maf* is upregulated in αGalCer-activated iNKT cells (48). *Runx1*-deficient mice showed a significant decrease in overall thymic iNKT counts, but showed only an iNKT17 deficit (47). Within *c-Maf*-deficient mice reduced *Ror*γ*t* expression and corresponding IL-17A production were found, but normal iNKT development (48). Both studies can be seen in favor of iNKT conversion, as essential iNKT TFs are required for iNKT17 differentiation and are not unique to iNKT17 cells.

### mTOR Effects on iNKT Development

Besides transcriptional regulation, the mTOR pathway has also been described to regulate iNKT cell fate. mTOR is a serine/threonine kinase, which regulates cell growth and metabolism. Two different mTOR complexes can be found: mTOR complex 1 containing Raptor, which is involved in “translation initiation, autophagy inhibition, lipid synthesis” (49) and control innate and adaptive immunity (50), and mTORC2 containing Rictor, which is “involved in actin remodeling and nutrient uptake” (49). Both pathways were shown to contribute to iNKT development, as iNKT cells frequencies were reduced in CD4^+^ T-cell-specific Raptor and Rictor conditional knockout mice (49, 50).

In CD4^cre^Raptor^fl/fl^ mice, the authors reported an accumulation of iNKT cells within stage 0, two-third in stage 1, one-third in stage 2, and an absent stage 3 (50). The remaining iNKT showed high PLZF expression, consistent with the early developmental block. Consistent with loss of stage 3 iNKT (iNKT1) cells, the authors also showed a decrease in T-bet expressing iNKT cells with concomitant enrichment of stage 1 iNKT (iNKT2) cells. However, the composition of stage 2 iNKT cells regarding iNKT2 and iNKT17 frequency was not fully elucidated.

Published literature is controversial regarding, which of the described iNKT subsets is affected in CD4^cre^Rictor^fl/fl^ mice. Two papers showed a cell intrinsic defect in iNKT cell development in the absence of Rictor (30, 49). However, while one group (49) demonstrated a substantial effect on NKT2 development and thymic IL-4 secretion and GATA3 expression, a second group reported a selective effect on the NKT17 lineage (30). The source of animals and the influence of the biomedical establishment on the microbiota could explain differences in the detection of NKT17 subset.

Of note, autophagy has also been described to play an essential role in iNKT cell development (51, 52). In T lymphocyte specific conditional knockout mice (CD4cre) lacking the essential autophagy genes Atg7 (51, 52) or Atg5 (52), iNKT cell development was blocked at an early stage and no mature peripheral iNKT cells were found (51, 52).

### Perspectives for Human iNKT Cell Therapy

Human and murine iNKT cells can both be divided into CD4^+^ and CD4^−^CD8^−^ cells, while human iNKT cells can also express CD8 (7). Human subpopulations are further characterized by CD161 (“equivalent to murine NK1.1”) expression (53). Human iNKT cells can be cytolytic (CD8^+^ and CD4^−^CD8^−^) (5), can produce T_H_1- (CD4^+^, CD8^+^, and CD4^−^CD8^−^) as well as T_H_2-type cytokines (CD4^+^) (5), and can secrete IL-17 in a pro-inflammatory environment (CD161^+^) (54). Human and murine iNKT cells are similarly activated (53), develop within the thymus (53), and depend on PLZF expression (24) despite their different surface receptor expression. Unlike murine iNKT cells, human iNKT cells are thought to leave the thymus in an immature state and mature within the periphery (53). As they produce the same cytokines, the underlining transcriptional mechanisms should be similar.

It is known that cell fates determine the overall direction of the immune response, for example, IFNγ production, seen in human NK, T-cells, and iNKT cells, is important for antitumor responses (55). Thus, increasing IFNγ-producing cells is one goal for tumor therapy. As iNKT cells—in contrast to CD3^+^ T-cells—have been shown to be unaffected by the suppressive effects of CD15^+^ granulocytic myeloid-derived suppressor cells in head–neck cancer patients (56), they represent an interesting tool for tumor therapy.

A recent Phase I clinical trial adoptively transferred iNKT cells into stage IIIB–IV melanoma patients after *in vitro* expansion with anti-CD3 and IL-2 proved to be safe and tolerable (57). Even though patient iNKT cells showed majorly enhanced IFNγ production posttreatment compared to pretreatment, they also produced IL-4 (57), which is associated with asthma (53, 58), and the anti-inflammatory cytokine IL-10 (57). As both cytokines can induce unwanted side effects within patients, understanding the molecular mechanisms during cell fate decisions could be beneficial for therapy. Thus, understanding transcriptional regulations within murine models can benefit human cancer therapies.

## Conclusion

Looking at these data within this review, one can find studies in favor of the developmental stage theory and studies against it. In favor of undergoing developmental stages is the distinct cut-off at stage 2 in *Erg2*-deficient mice (34) and negative regulatory genes control of iNKT17 development (27, 40), which could also be seen as a separation from iNKT2 cells occupying stage 2. Also, CD4^cre^Raptor^fl/fl^ mice showed an accumulation of iNKT cells within stage 0 and a reduction in stage ½ (50). All these studies suggest a shared developmental pathway within iNKT cells. In favor of lineage differentiation is the increased iNKT17 population in SAP-deficient mice with normal iNKT1 cell counts and an absent iNKT2 population (25) and the observation that TCR signaling strength as seen in *Fc*ε*R1*γ-deficient mice might give rise to one population instead of another (27). Further, if a shared developmental stage is assumed, iNKT2 and iNKT17 sharing stage 2 should cluster more within the PCA (28, 31).

All in all, murine iNKT cell development still seems to be puzzling. Overall some differences in iNKT subset detection may be semantic and depends on individual mouse strain used. Furthermore, microbial effects in mice within different breeding facilities may influence different iNKT subset composition seen within different publications. Nevertheless, more insight will be gained by deeper transcriptional analyses parallel to phenotyping, as these analyses are currently limited to 20 fluorophores. Unbiased approaches such as Cytof or tSNE may further reveal iNKT cell differences and may account for the observed mouse strain specific differences. Furthermore, both approaches can reveal more insights into human iNKT cell development and highlight how these cells can be used more effectively in cancer therapy.

## Author Contributions

SB has designed, written, revised, and approved of the review herself. She is accountable for all aspects in this review.

## Conflict of Interest Statement

The author declares that the research was conducted in the absence of any commercial or financial relationships that could be construed as a potential conflict of interest.

## Abbreviations

CD, cluster of differentiation; CDR, complementarity-determining regions; convT-cell, conventional T-cells; DP, double-positive; FACS, fluorescence-activated cell scanning; GATA-3, GATA-binding protein 3; GM-CSF, granulocyte macrophage colony-stimulating factor; IFNγ, interferon gamma; IgE, immunoglobulin E; IL, interleukin; IL-XR, interleukin X receptor; ILCs, innate lymphoid cells; iNKT cells, invariant natural killer T-cells; MAIT, mucosal-associated invariant T-cells; MHC, major histocompatibility complex; MIR, modulator of immune recognition; miRNA, MicroRNA; mTOR, mammalian target of rapamycin; mTORC, mTOR complex; NK, natural killer; NKAP, NFKB activating protein; NKT cells, natural killer T-cells; NKT_II cells, type II NKT cell; PBMC, peripheral blood mononuclear cell; PCA, principle component analyses; pLN, peripheral lymph nodes; PLZF, promyelocytic leukemia zinc finger; RORγT, RAR-related orphan receptor gamma; SAP, SLAM-associated protein; SLAM, signaling lymphocytic activation molecule; TCR, T-cell receptor; TF, transcription factors; UTR, untranslated region; wt, wildtype.
